# 17β-Oestradiol Protects from Hepatitis C Virus Infection through Induction of Type I Interferon

**DOI:** 10.3390/v14081806

**Published:** 2022-08-18

**Authors:** Matteo Nazzareno Barbaglia, James Michael Harris, Artem Smirnov, Michela Emma Burlone, Cristina Rigamonti, Mario Pirisi, Rosalba Minisini, Andrea Magri

**Affiliations:** 1 Department of Translational Medicine, Università del Piemonte Orientale, 28100 Novara, Italy; 2 Nuffield Department of Medicine, University of Oxford, Oxford OX3 7FZ, UK; 3 Ludwig Institute for Cancer Research, University of Oxford, Oxford OX3 7DQ, UK

**Keywords:** oestrogen, hepatitis C, interferon

## Abstract

Background and Aims: Sex hormones are widely recognised to act as protective factors against several viral infections. Specifically, females infected by the hepatitis C virus display higher clearance rates and reduced disease progression than those found in males. Through modulation of particle release and spread, 17β-oestradiol controls HCV’s life cycle. We investigated the mechanism(s) behind oestrogen’s antiviral effect. Methods: We used cell culture-derived hepatitis C virus in in vitro assays to evaluate the effect of 17β-oestradiol on the innate immune response. Host immune responses were evaluated by enumerating gene transcripts via RT-qPCR in cells exposed to oestrogen in the presence or absence of viral infection. Antiviral effects were determined by focus-forming unit assay or HCV RNA quantification. Results: Stimulation of 17β-oestradiol triggers a pre-activated antiviral state in hepatocytes, which can be maintained for several hours after the hormone is removed. This induction results in the elevation of several innate immune genes, such as interferon alpha and beta, tumour necrosis factor, toll-like receptor 3 and interferon regulatory factor 5. We demonstrated that this pre-activation of immune response signalling is not affected by a viral presence, and the antiviral state can be ablated using an interferon-alpha/beta receptor alpha inhibitor. Finally, we proved that the oestrogen-induced stimulation is essential to generate an antiviral microenvironment mediated by activation of type I interferons. Conclusion: Resulting in viral control and suppression, 17β-oestradiol induces an interferon-mediated antiviral state in hepatocytes. Oestrogen-stimulated cells modulate the immune response through secretion of type I interferon, which can be countered by blocking interferon-alpha/beta receptor alpha signalling.

## 1. Introduction

It is well known that sex is one the most important factors affecting the natural history of the hepatitis C virus (HCV) infection. Specifically, female sex acts as a protective factor, favouring higher spontaneous viral clearance rates following acute infection [1,2]. Moreover, disease progression is slower in females compared to males, who have a 10-fold higher rate of progression to advanced fibrosis irrespective of age [3]. However, liver disease progression is not linear across different stages of a woman’s life; a different progression rate can be observed among females during the pre- and postmenopausal periods [4,5]. In response to the HCV, premenopausal women show reduced inflammation and liver disease progression, in contrast to their postmenopausal counterparts [6,7,8].

The major discriminant factor between pre and postmenopausal status is sex hormone levels, and these have been described as protective agents against several pathogens, including RNA viruses, such as SARS-CoV-2 [9,10], influenza [11] and HIV [12]. Oestrogens are amongst the best studied sex hormones, and the intricate connection between oestrogens and host antiviral systems is highlighted by their ability to modulate both innate and adaptive immune cells, which makes them paramount to counteracting viral infections (reviewed in [13,14]). These findings suggest that hormonal factors, specifically oestrogens, may exert a protective effect on the HCV’s disease progression [15].

Previous in vitro studies have demonstrated that 17β-oestradiol (E2) triggers an antiviral state in hepatocytes, capable of perturbing HCV’s assembly and release [16] and modulating viral entry [17]. This underlines a central role for these cells in the association between hormonal milieu, viral clearance and liver disease progression. The antiviral activity of E2 is mediated by activation of the oestrogen receptors alpha/beta (ERα/β) or the G protein-coupled receptor 30 (GPR30) [16,17], but our understanding of the oestrogen-induced antiviral pathways in hepatocytes remains unclear.

Here, we aim to investigate the mechanism(s) behind the oestrogen-mediated antiviral status, evaluating the impact of oestrogen signalling on intrinsic hepatocyte defences.

## 2. Materials and Methods

### 2.1. Cell Cultures and Chemical Compounds

The human hepatoma-derived HuH7 [18] cells were grown in DMEM (1X) + GlutaMAX™-I, supplemented with 10% foetal bovine serum (FBS), 100 U/mL penicillin, 100 μg/mL streptomycin, 0.1 M non-essential amino acids, 25 mM HEPES (N-2-hydroxyethylpiperazine-N-2-ethane sulfonic acid) and 1 mM sodium pyruvate (Thermo Fisher Scientific, Milan, Italy). According to different models, HuH7 cells were exposed to different concentrations of 17β-oestradiol (E2) (400 or 200 nM) (Sigma-Aldrich, Milan, Italy), fulvestrant (400 nM) (Sigma-Aldrich, Milan, Italy), IFN alpha-IFNRA-IN-1 (IFNARi) (500 nM) (MedChemExpress, Monmouth Junction, NJ 08852, USA) or interferon alpha-2a (IFNα-2a) (10^3^–10^−4^ MUI/mL) (Roche S.P.A, Monza, Italy).

### 2.2. HCV Infection

Infection was performed using the HCV strain JFH-1 (genotype 2a) at a multiplicity of infection (MOI) of 0.1. Cells were infected in the presence or absence of treatments for 3 h according to the experimental model. Later, the inoculum was removed, and media were replaced. The antiviral efficacy was evaluated by focus-forming unit assay (FFU) or by intracellular HCV RNA quantification, as previously reported [16].

Extracellular viral particles were measured by viral titration using the FFU assay (FFU/mL): HuH7 cells were infected for 3 h with serial dilutions of the collected infective supernatants. After the viral inoculum was replaced with the fresh media for 72 h, the viral titre was evaluated by FFU count.

### 2.3. Re-Sensitising to Infection

HuH7 cells were exposed to E2 according to two different models: short-term and long-term. In the short-term model, HuH7 cells were incubated for 16 h (overnight) with two different E2 concentrations (400 and 200 nM) or DMSO (control group). Subsequently, the treatment was removed and replaced with fresh media for different time points (0, 2, 3, 4, 5, 6 and 24 h) before HCV infection.

In the long-term model, HuH7 cells were maintained in culture for 14 days using media supplemented with E2 (400 nM) or DMSO. Treatment was then removed and replaced with the fresh media for 0, 1, 2, 3, 4 and 5 days before HCV infection. In both models, cells were maintained for 72 h post infection and the antiviral activity was evaluated by FFU assay.

### 2.4. Time-Course Expression Analysis

HuH7 cells were exposed to E2 (400 nM) or DMSO for 4 h (E2-treated cells and naïve cells, respectively) and then maintained in fresh media. Samples were harvested at different time points (0, 6, 24, 48 and 72 h) using NucleoZOL (MACHEREY-NAGEL, Bethlehem, PA, USA) for subsequent RNA extraction. For the infection model, HuH7 cells were treated with E2 or DMSO for 1 h, before a 3 h infection in the presence of respective treatments. Samples were collected as described above.

### 2.5. RNA Extraction, RT-qPCR and Gene Expression Analysis

Total RNA was extracted from the aqueous phase following the manufacturer’s protocol and quantified by Nanodrop (Thermo Fisher Scientific, Milan, Italy). A total of 250 ng of total cellular RNA was reverse-transcribed using the High-Capacity cDNA Reverse Transcription Kit (Thermo Fisher Scientific, Milan, Italy) and amplified by qPCR using the Power SYBR™ Green Master Mix (Thermo Fisher Scientific, Milan, Italy) with specific primers (Table S1) or using TaqMan™ Gene Expression Master Mix with TaqMan probes (Table S2) (Thermo Fisher Scientific, Milan, Italy). Hypoxanthine-guanine phosphoribosyltransferase (*HPRT*) was used as housekeeping gene, and the relative gene expression, normalised to naïve cells (untreated and uninfected), was determined using the 2^−ΔΔCt^ method.

### 2.6. Conditioned Media Production

HuH7 cells were treated with E2 (400 nM) or DMSO for 4 h. Subsequently, cells were washed thrice with PBS, and media were replaced. The respective conditioned media (CM) was collected at 48 or 72 h post treatment and stored at −80 °C until use.

### 2.7. Combination Treatment Models

The antiviral activities of E2, CM and IFNα-2a were tested in combination with IFNARi (500 nM) following the subsequent models:

E2 + IFNARi: HuH7 cells were treated with E2 for 1 h, followed by infection with HCV in the presence of E2. FFU assay and HCV RNA quantification were performed at 72 h post infection. Based on the experimental model, IFNARi was added for the whole time of the experiment or at specified times post infection (0 ÷ 72, 24 ÷ 72 and 48 h ÷ 72).

CM + IFNARi: HuH7 cells were treated for 1 h with the respective CM collected at 48 or 72 h (CM_48_ or CM_72_) with or without IFNARi. Infection was performed for 3 h in the presence of specified treatments. Subsequently, the viral inoculum was removed, and media were replaced. Seventy-two hours later, supernatants were collected, and viral titre (FFU/mL) was evaluated.

IFNα-2a + IFNARi: HuH7 cells were infected for 3 h, and then cells were cultured for 72 h in the presence of serial dilutions (1:10) of IFNα-2a (10^3^–10^−4^ MUI/mL) or DMSO, with or without IFNARi. Viral infection was evaluated by FFU assay.

### 2.8. Statistical Analyses

Statistical analyses were performed using Prism 9.3 (GraphPad, La Jolla, CA, USA). Data are shown as means ± SEM; probabilities are indicated by *p* values obtained by the indicated test. When 2 groups were compared with multiple *t*-tests, *p* values were corrected according to Bonferroni’s method. When multiple groups when compared with a Kruskal–Wallis one-way analysis of variance, *p* values were adjusted and corrected with Dunn’s method. The threshold for statistical significance was 0.05 (two-tailed). Bubble plots and line plots were created in R using the ggplot2 package.

### 2.9. Bioinformatics

A gene set enrichment analysis was performed using the Hallmark gene sets from the Molecular Signatures Database in GSEA_v4.1. Affymetrix microarray datasets from the HCV (GSE14323) and HBV (GSE83148) cohorts were accessed from the Gene Expression Omnibus. GSEA was run using 50,000 gene set permutations.

## 3. Results

### 3.1. Oestrogen Exposure Induces Interferon Signalling

**Figure 1:**
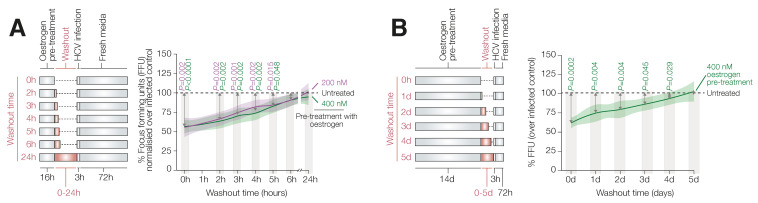
Re-sensitising to infection. (**A**) HuH7 cells were treated overnight with 2 doses of oestrogen and maintained from 0 to 24 h in fresh media (Washout) before infection. Cells were then incubated in fresh media. (**B**) HuH7 cells were treated with oestrogen for 14 days and then maintained from 0–5 days in fresh media (Washout) before infection. Cellular susceptibility to infection was evaluated by FFU count. Data represent at least 3 independent experiments with 2 biological replicates. Values were normalised to the untreated control (dashed line) and presented as mean ± SEM. Statistical significance was evaluated by Mann–Whitney U test.

Initially, we evaluated the persistence of the oestrogen-mediated antiviral status after the removal of receptor stimulation and its impact on cellular susceptibility to infection. For this purpose, HuH7 cells were exposed to E2 (200 or 400 nM) overnight, and then oestrogen-conditioned media were replaced with fresh media. At that point, different batches of cells were infected at time points ranging from 0 to 6 h or 24 h later. We observed a time-dependent reduction in antiviral activity with an estimated 50% decrease by around 4 h; a full recovery of the infectivity was observed after 6 h for both of the oestrogen concentrations tested (Figure 1A). We then investigated the protective role of oestrogen in a long-term culture. For this purpose, HuH7 cells were cultured in the presence of 400 nM of E2 for 14 consecutive days and then re-sensitised with normal media for up to 5 days before infection. In line with what occurred in the short exposure model, we observed a time-dependent increase in viral susceptibility with a 50% recovery at 2.4 days and a full recovery after 3 days (Figure 1B).

**Figure 2:**
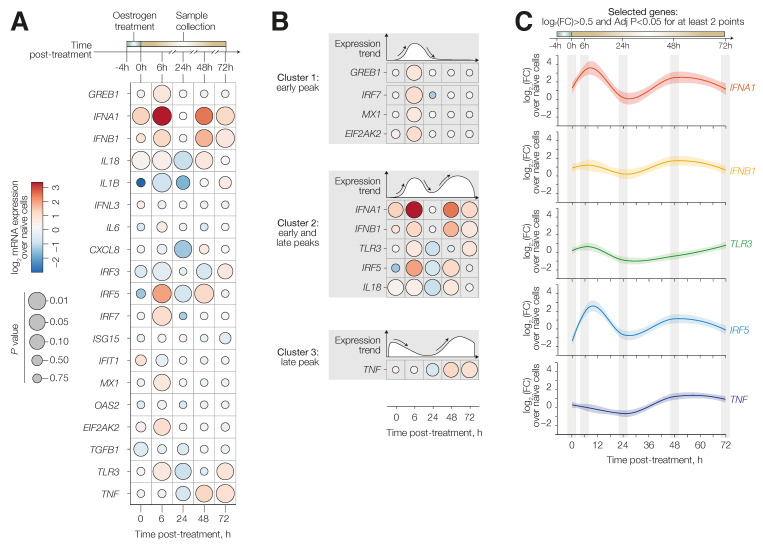
Gene expression profile of E2-treated cells. HuH7 cells were treated with 17β-oestradiol or DMSO (naïve cells) for 4 h and then incubated in fresh media. Gene expression was evaluated at different time points as indicated. The mRNA levels are reported as log_2_ (FC) normalised to naïve cells. (**A**) Bubble plot of the mRNA fold-change from 18 innate immune genes in oestrogen-treated cells. Data represent the mean of at least 3 independent experiments with 2 biological replicates. Statistical significance was evaluated by multiple *t*-tests with Bonferroni correction. (**B**) Grouping of the three distinct clusters based on their expression pattern: early (**top**), early and late (**middle**) and late (**bottom**). (**C**) Time-course gene expression of the five genes with log_2_ (FC) > 0.5 and *p* < 0.05 for at least two time points. In each graph, the line represents the gene expression trend based on mean values for each time point. Shading shows SD.

Since it has been previously described that 17β-oestradiol can induce interferon signalling during viral infections [19,20], we hypothesised that the oestrogen-mediated antiviral status could be ascribed to pre-activation of interferon signalling-pathways. As it has been reported that E2 is capable of inducing an early and late transcriptional response depending on the target genes [21], HuH7 cells were treated with E2 (400 nM) for a reduced period of 4 h, and samples were collected at 0, 6, 24, 48 and 72 h post treatment. We then analysed the mRNA expression levels of 18 immuno-response genes (Tables S1 and S2) alongside growth regulation by oestrogen in breast cancer 1 (*GREB1*), which has been reported to be an oestrogen receptor-regulated gene [22] (Figure 2A). Differential gene expression analysis identified nine genes which were significantly upregulated at at least one time point. We noted that those genes could be divided into three separate clusters according to their expression kinetics: early peak (cluster 1) with an upregulation between 0 and 6 h, early and late peaks (cluster 2) with elevated expression between 0 and 6 h and after 48 h; late peak (cluster 3) with an upregulation after 48 h (Figure 2B). Specifically, we found a significant upregulation of eight genes belonging to cluster 1 or 2 after 6 h (*IFNA1*, *IFNB1*, *TLR3*, *IRF5*, *IRF7*, *MX1, IL18* and *EIF2AK2*), which are associated with elevated levels of the *GREB1* control gene (Figure S1). Overall, we observed reduced expression between 6 to 24 h, noting significant downregulation of *CXCL8*, *IL18*, *IRF5* and *IL1B*. We detected a subsequent upregulation of *IFNA1*, *IFNB1*, *IL18*, *IRF5* and *TNF* (cluster 2 or 3) between 24 and 48 h. Increased expressions of *IFNA1*, *IFNB1*, *TLR3* and *TNF* were also observed at the 72 h time point (cluster 2 or 3) (Figure 2B). Based on these data, we focused our analyses on a gene-set consisting of five genes: *IFNA1*, *IFNB1*, *TNF*, *IRF5* and *TLR3*, which were significantly elevated (Log_2_ (FC) > 0.5; adjusted *p* value < 0.05) at at least two separate time points (Figure 2C). Overall, the kinetic expression profiles suggested that an oestrogen-mediated antiviral status induces a first peak of interferon signalling between 0–6 h post treatment and a refractory period at around 24 h, followed by a second, prolonged period between 48 and 72 h.

### 3.2. Oestrogen Antiviral Activity Is Type I Interferon-Mediated

**Figure 3:**
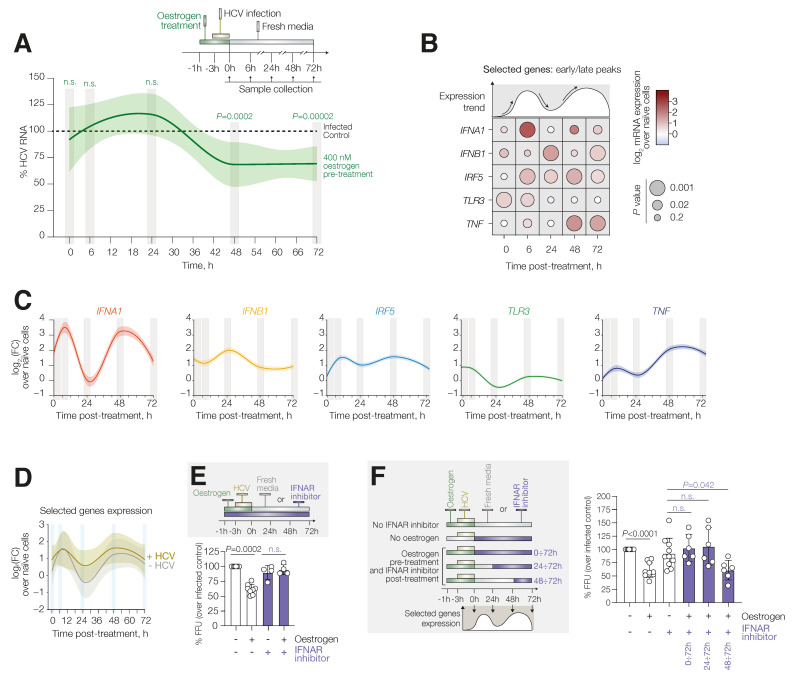
E2-mediated antiviral effect is type I interferon dependent and not affected by viral replication. HuH7 cells were treated with 17β-oestradiol or DMSO (infected control) for 1 h, followed by infection in the presence of inhibitor treatment. HCV RNA quantification and gene expression analysis were performed as indicated. (**A**) Intracellular HCV RNA (%) of oestrogen-treated cells relative to infected and untreated control (dashed line). Data represent the mean of at least 3 independent experiments with 2 biological replicates. Statistical significance was evaluated by Mann–Whitney U test. (**B**,**C**) Bubble plot (**B**) or time-course gene expression (**C**) of the five genes. Data represent the mean mRNA fold change (log_2_ (FC)) in oestrogen-treated and infected cells normalised and compared to naïve cells. Data are derived from at least 3 independent experiments with 2 biological replicates. Statistical significance was evaluated by multiple *t*-tests with Bonferroni correction. (**D**) Time-course gene expression comparison of the 5-gene set between oestrogen-treated cells with or without HCV infection. Data represent the mean mRNA fold change (log_2_ (FC)) in oestrogen-treated cells, with or without infection, normalised to naïve cells. Data are derived from at least 3 independent experiments with 2 biological replicates. Statistical significance was evaluated by Wilcoxon signed-rank test. (**E**,**F**) HuH7 cells were treated with 17β-oestradiol for 1 h, followed by infection in the presence of oestrogen. IFNAR inhibitor was added for the entire duration of the experiment (**E**) or added at different time points post infection (0 ÷ 72 h, 24 ÷ 72 h and 48 ÷ 72 h) (**F**). FFU assay were performed at 72 h post infection and results were normalised to untreated control cells. Data represent the mean and SEM of at least 3 independent experiments with 2 biological replicates. Statistical significance was evaluated by Mann–Whitney U test. n.s.: not significant.

Based on previous results, we investigated whether the oestrogen-mediated activation of interferon signalling could be maintained in the presence of an HCV infection and have implications for the antiviral effect. To explore this, we pre-treated HuH7 cells with E2 (400 nm) for 1 h before infection with HCV for 3 h while maintaining E2 in the media. The inoculum was then removed, and the media were replaced with samples collected at 0, 6, 24, 48 and 72 h post infection. HCV RNA replication was monitored at each time point by qPCR. As expected, we observed a significant reduction in HCV RNA in the presence of E2 treatment with a 25% and 31% decrease at 48 and 72 h post infection, respectively, whilst no effect was observed in the first 24 h (Figure 3A). We then quantified the expression of the previously identified five-gene set and found that in the presence of viral infection E2 treatment may still induce the expression levels of the five genes compared to naïve cells. Specifically, we observed a significant induction of *IFNA1* at 6, 48 and 72 h and an elevation of *IRF5* at 6, 24, 48 and 72 h. We also detected increased levels of *TLR3* at 0 and 6 h and of *TNF* at 6, 48 and 72 h (Figure 3B). Importantly, in four of the selected genes (*IFNA1*, *IRF5*, *TLR3* and *TNF*), we still observed the “2-peak” phenotype we reported in E2 treatment alone, showing overall induction at 6 and 48 h post treatment. However, we noted that *INFB1* was elevated at 0, 24 and 72 h, showing a mid-peak profile (Figure 3C). Despite perturbation of *IFNB1* expression by HCV infection, it is important to note that we found no significant differences in the global expression of the gene set between E2 alone and E2 in the presence of HCV at any time point, whether *IFNB1* is included (Figure 3D) or excluded from the gene set (Figure S2). These data suggest that E2 stimulation induces an interferon-mediated response independent of active viral replication.

To confirm that the E2-mediated antiviral state is dependent on type-1 interferon signalling, HuH7 cells were pre-treated with E2 for 1 h in the presence of the “inhibitor of the interaction between IFN-α and its receptor (IFNAR)” (IFNARi) [23] at 500 nM. We demonstrated that this concentration was sufficient to reduce interferon activity (Figure S3). Following infection, in the presence of both E2 and IFNARi, cells were exposed to IFNARi for a further 72 h to abrogate both of the previously identified signalling peaks. The oestrogen-mediated antiviral effect of approximately 40% on HCV infectivity (as % FFU) and HCV RNA was completely reverted by IFNARi, showing comparable HCV levels to control cells (Figure 3E and Figure S4). Based on our previous results showing the time-dependency of the oestrogen-mediated interferon induction, we investigated the role of each interferon peak on the anti-HCV activity (6 vs. 48 h). We treated HuH7 cells with E2 (400 nM) for 1 h and infected them in the presence of E2 for 3 h. After infection, a fresh medium containing IFNARi was added at 0, 24 or 48 h post infection. Adding IFNARi immediately post infection (0 h) resulted in a block of both peaks with unimpeded viral replication; the same happened when the IFNARi was added 24 h p.i., corresponding to the refractory period after the first peak. In contrast, adding IFNARi at 48 h p.i. (during the second peak) resulted in viral levels similar to those observed in E2-treated cells not exposed to the IFNARi (Figure 3F), confirming that the antiviral response is triggered during the early induction period. Collectively, these data suggest that the type I interferon response is the key regulator of oestrogen-mediated antiviral activity.

### 3.3. Oestrogen Antiviral Activity Is Mediated through Released Cytokines

**Figure 4:**
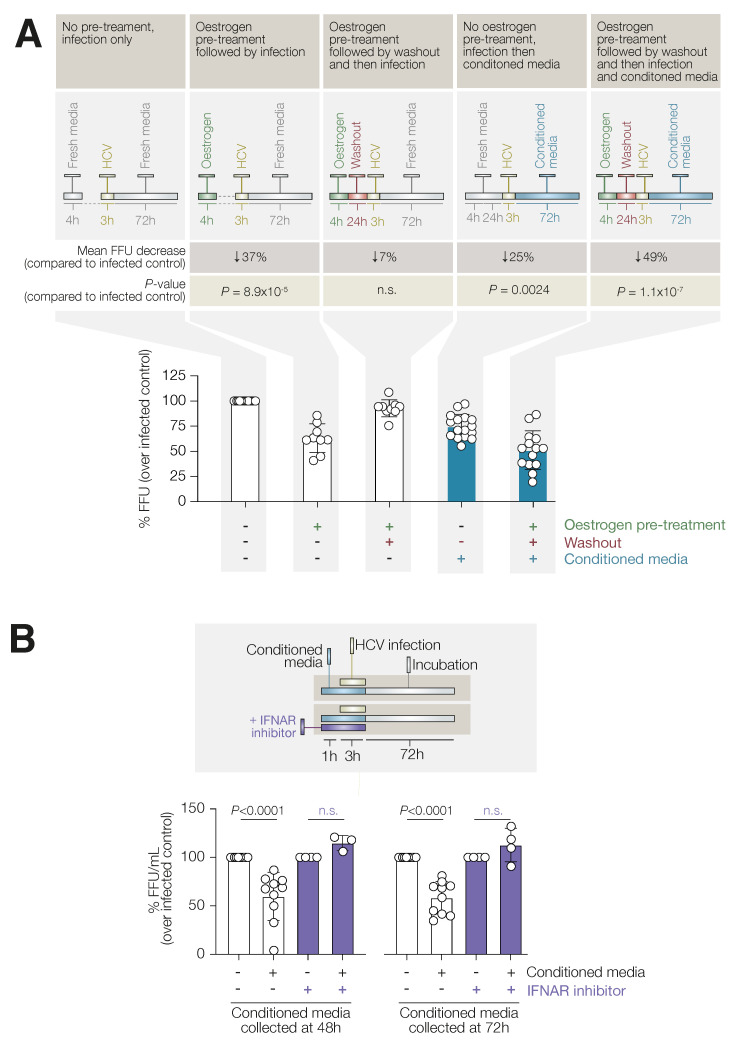
Oestrogen-conditioned supernatant is essential for antiviral activity. (**A**) HuH7 cells with or without 4 h oestrogen pre-treatment were infected with HCV. Infection occurred either immediately post-oestrogen treatment or following a 24 h of washout period with fresh media. Infected cells were then cultured in either conditioned media collected at 24 h or fresh media. Antiviral activity was evaluated at 72 h post infection by FFU assay. Statistical significance was evaluated by Kruskal–Wallis one-way analysis of variance with Dunn’s *p* value correction. (**B**) HuH7 cells were treated with CM_48_ or CM_72_ for 1 h with or without IFNARi, followed by infection in the presence of indicated treatments. Cells were then incubated in fresh media for 72 h, and the extracellular viral titre (FFU/mL) was evaluated through FFU assay. Data represent the mean and SEM of at least 3 independent experiments with 2 biological replicates. Statistical significance was evaluated by Mann–Whitney U test. n.s.: not significant.

Based on our findings, we hypothesised that oestrogen-stimulated cells may secrete molecules capable of inducing an antiviral status. To explore this possibility, Huh7 cells were treated with E2 for 4 h before re-sensitisation in fresh media for a 24 h period, after which conditioned media (CM) were collected. Cells were then infected for 3 h and incubated in either normal or conditioned media for a further 72 h. As expected, a 24 h re-sensitisation resulted in a full recovery of viral infection, as observed for the longer E2 treatment in Figure 1 (Figure 4A, third column). We then assessed whether the CM alone were able to mediate an antiviral response or if a direct oestrogen stimulation was needed. HuH7 cells with or without E2 treatment (4 h) were infected with HCV after the 24 h re-sensitisation and then exposed to the CM for 72 h. Interestingly, the CM in the absence of E2 pre-treatment were sufficient to induce a 25% reduction in HCV replication (Figure 4A, fourth column), although E2 pre-treatment induced a more potent suppression of viral replication (49%) (Figure 4A, fifth column), confirming that an oestrogen-induced pre-activation status is essential to maximise the antiviral effect.

To investigate whether any antiviral effect mediated by a secreted factor could be ascribed to type-I IFNs and IFNAR interactions, naïve HuH7 cells were treated with E2 (400 nM) for 4 h, followed by a 48 h or 72 h incubation in fresh media. After which, the conditioned media were collected (CM_48_, CM_72_). We pre-treated naïve HuH7 cells with CM_48_ or CM_72_ for 1 h, followed by a 3 h infection with HCV still in the presence of conditioned media, with or without IFNARi, to ablate any type-I interferon signalling. We then titrated the viral particles released in the media and found that both CM_48_ and CM_72_ were able to suppress HCV infectivity by approximately 50%, similar to direct E2 stimulation. In contrast, IFNARi was able to fully recover viral infectivity (Figure 4B), further demonstrating the role of type I interferon signalling in oestrogen-mediated antiviral activity.

### 3.4. Biological Sex Confers Endogenous Innate Immune Protection

**Figure 5:**
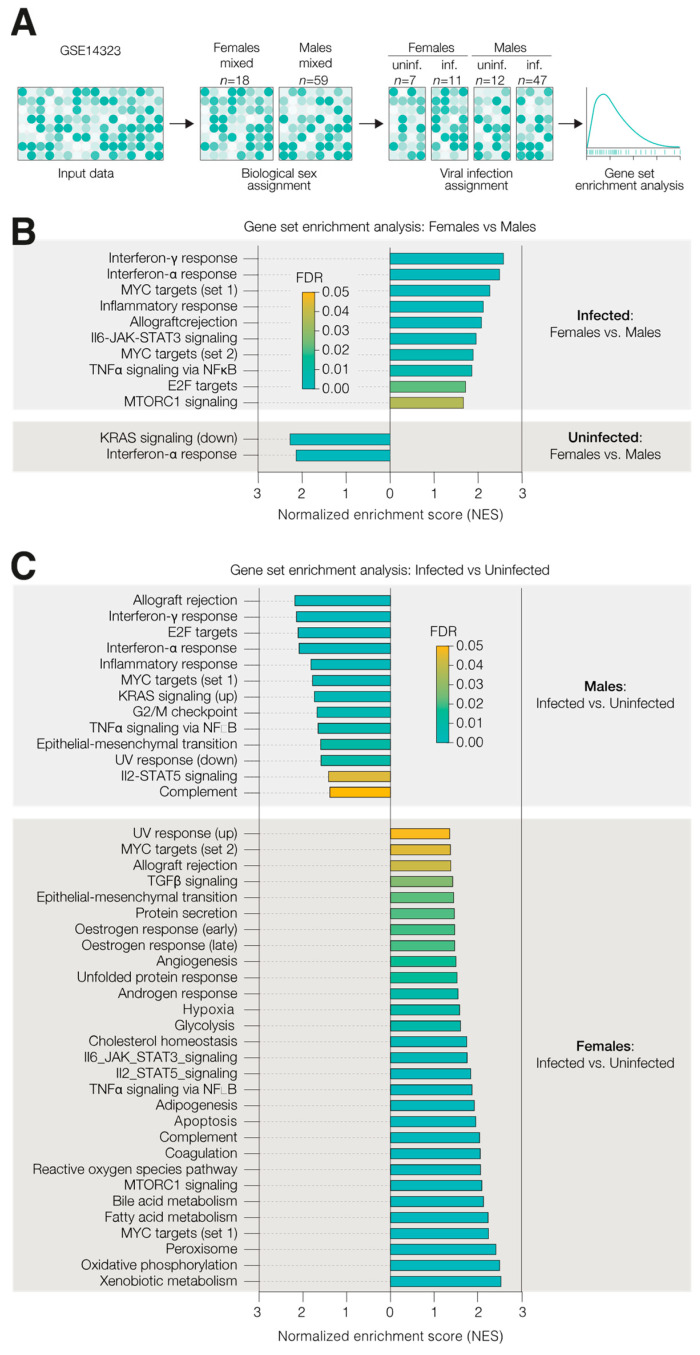
Biological sex confers endogenous innate immune protection. (**A**) Using GSEA, Affymetrix microarray data were interrogated to understand the impact of biological sex on host cell processes. (**B**) Hallmark gene set expression was assessed to show the sex-specific pathway enrichment in the presence (**top**) or absence (**bottom**) of HCV infection. Hallmarks showing significant enrichment in females are plotted, ranked by normalised enrichment score, and coloured based on FDR. (**C**) Separating patients by biological sex, we applied GSEA to understand the impact of viral infection on the hepatic transcriptome.

To expand our in vitro observations, we explored the relationship between biological sex and inflammatory pathways in a cohort of HCV positive patients [24]. We obtained publicly available Affymetrix microarray data from patients with virally induced liver cirrhosis or HCC, as well as control subjects, with no record of HCV- or alcohol-associated liver damage, although there was no record of biological sex associated with this cohort. Using a previously published set of X and Y chromosome-associated hepatic gene transcripts [25], we assigned biological sex to the patients using their expressions of four genes (*EIF1AY, RPS4Y1, KDM5D* and *XIST*) (Figure S5A, left). We confirmed that the expression of these genes was sufficient to discern between sexes in an independent cohort of chronic hepatitis B patients [26] and noted similar expression levels and separation between males and females (Figure S5A, right). Using a gene det enrichment analysis (GSEA) and the Hallmark gene sets, we assessed that the cellular processes that were differentially enriched in males and females in the presence or absence of an HCV infection (Figure 5A). Ordinarily, GSEA is performed using an exploratory FDR value of 25%; however, to increase the statistical stringency of our analyses, we used a conservative FDR of 5%. Consistent with previous reports, we observed that female subjects exhibit evidence of innate immune activity above male subjects, irrespective of their HCV status (Figure 5B). Infected females show an enrichment of genes associated with both interferon alpha and gamma activity, as well as NF-KB and STAT signalling pathways (Figure 5B). Importantly, we show that there is no sex specific expression pattern of our five gene signature (Figure S5B).

To assess the contribution of HCV to the hepatic transcriptome, we assessed the processes that were enriched in infected patients above the non-viral controls of each sex. Our analyses reveal that the number of pathways upregulated in females vastly outnumbers those that are responsive in males and represent gene sets involved in a wide range of cellular functions. However, we only note an upregulation of interferon alpha- and gamma-associated genes in infected male patients, suggesting that females might exhibit a naturally elevated inflammatory response due to the presence of endogenous oestrogens (Figure 5C).

## 4. Discussion

Our experiments show that E2-mediated antiviral activity is exerted through an activation of hepatocyte innate immunity and expression of type I interferon, in a time-dependent manner. These findings need to be interpreted in the light of the vast literature concerning the interaction between sex hormones and viral infection. In fact, oestrogens have been demonstrated to inhibit the replication of several viruses, including SARS-CoV-2 [9], influenza [11], HBV [27] and HTLV [28] and to modulate EBV [29] and HIV [30] latency. With regards to HCV, 17β-oestradiol has been documented to inhibit its life cycle through distinct mechanisms: it can modulate HCV entry by GPR30 activation, reducing the levels of the HCV receptor occludin [17], and, as previously reported by our group, it can reduce HCV’s release and spread, through its nuclear oestrogen receptor alpha/beta (ERα/β) [16].

We initially demonstrated that E2’s antiviral restriction on an HCV infection can be maintained for several hours after direct hormonal stimulation has been removed. We showed, in the short-term model, that full viral recovery occurs at 6 h, whilst in the long-term model 5 days were required to restore full viral susceptibility. These results are consistent with the in vivo data, which show that E2 has a prolonged half-life, ranging from 2 h to 10 days, depending on the route of administration [31,32,33,34].

We then evaluated the effect of E2 stimulation on a panel of innate immune genes and identified a set of five genes that were induced by oestrogen treatment; of note, the most significant induction was observed in the IFNA1, IFNB1 and TNF mRNA levels. This observation is in line with previous findings demonstrating that E2 protects macrophages from HIV infection through induction of type I IFNs [19]. It has also been previously documented that E2 can induce type I interferon production, through direct or indirect induction of IFN genes by oestrogen nuclear receptor activation [35,36,37].

Our experiments identified TNF as a member of our five-gene signature; TNF is a potent cytokine and an important mediator of cell signalling during inflammation [38]. E2-mediated TNF activation has been documented to suppress HTLV through tax activation [28]. TNF and IFN signalling have also been previously connected in the context of HCV and HEV infections by Wang et al., showing that TNF signalling induces the production of several interferon-stimulated genes [39]. The convergence of these pathways leads to an enhanced antiviral response, leading to the control of viral spread, consistent with our results. Importantly, TNF has been documented to suppress HCV’s spread [40], recapitulating the E2-mediated antiviral phenotype.

We also report that the five-gene set induction showed a two-peak profile at different time points (6 and 48 h), which was neither enhanced nor altered by viral infection. We further validated our observation by demonstrating that E2’s antiviral activity is mediated by IFNα/IFNAR interaction and can be rescued using an inhibitor that fully reverses the viral phenotype. It can be argued that the two-peak pattern reflects the refractory period known as hypo-responsiveness to re-stimulation, as reported previously [41]. Furthermore, it has been demonstrated in vivo that persistent stimulation induces multiple interferon peaks of similar intensities instead of a single peak [42], which provides in vitro support for our findings. The complex interplay between oestrogen and interferon signalling has been investigated by Panchanathan and colleagues, who showed that sustained IFN stimulation could transcriptionally modulate ERα mRNA and protein levels through STAT1, uncovering a novel feedback loop between oestrogen and interferon signalling pathways [43]. The two-peak model we observed in our experimental conditions is consistent with these data.

We then showed that E2-mediated antiviral activity is determined by secretion of cytokines that are able to activate IFNAR in a time-dependent manner. These data are supported by previous studies which suggest that E2 can promote the production of type I interferons through the nuclear receptor ERα [14]. Importantly, Lee et al. also reported that HCV can trigger an antiviral response through autocrine stimulation [44], which is compatible with our observations. Furthermore, it has been demonstrated that E2 can induce IFN gamma (IFNγ) transcription, through direct binding of an oestrogen-responsive element (ERE) in its promoter [45].

Finally, we report that females have a naturally elevated interferon response compared to males, and we observed that this phenotype was independent of viral infection. Our observation is in complete agreement with Marchi et al., who showed that females have an increased interferon response compared to that of males; they also showed how the interferon lambda 4 (IFNL4) genotype associated with the female sex may lead to an exaggerated interferon response [46]. Although we cannot discern a potential role for disease status, our observation is consistent with several reports that show the activation of dendritic cells in females leads to increased production of type-I interferon and TNF [47,48]. Furthermore, it has been demonstrated that in postmenopausal women 17β-oestradiol treatment could induce IFNα production in dendritic cells via TLR7 and TLR9 [49]. Previous studies have reported that E2 can regulate the production of IFNγ in vitro [50], and oestradiol can regulate type I and II IFNs production in systemic lupus erythematosus [51]. Further validation of oestrogens that regulate innate immunity has been provided by Jansen et al., who showed that innate immune gene expression differs between pre- and postmenopausal women [52]. Consistent with these observations, it has been shown that IFN pathway genes are expressed in higher abundance in women than men, and they can reach even higher induction after IFN stimulation [35,37].

Although we uncovered a role for E2 to restrict HCV’s replication through regulation of innate immunity in liver cells, it is known that oestrogens regulate a plethora of cellular functions. Importantly, ERα has been reported to regulate lipid metabolism and gluconeogenesis in hepatocytes [53], suggesting that an E2-mediated antiviral effect could be exerted through additional mechanisms. Furthermore, it is important to note that the liver is comprised many different cell types, including the non-parenchymal liver cells (Kupffer cells (KCs), hepatic stellate cells (HSCs) and liver sinusoidal endothelial cells (LSECs) amongst other immune cells. Since we demonstrated that the E2-mediated antiviral effect is modulated by secreted molecules, it is possible to speculate that these molecules could modulate the behaviour of cell types other than hepatocytes. Indeed, the antiviral response against HCV infection is a complex process, which is orchestrated by the interaction of the hepatocytes with other cell types [54].

In conclusion, we present a novel role for 17β-oestradiol in controlling HCV infection through modulation of type I interferon in hepatocytes. We demonstrate that the antiviral properties of E2 are not limited to intracellular signalling and can stimulate additional extracellular cell responses.

## Data Availability

Liver tissue data were obtained from GSE14323.

## Supplementary Materials

The following supporting information can be downloaded at: https://www.mdpi.com/article/10.3390/v14081806/s1, Figure S1: E2 stimulation increases the level of control gene GREB1; Figure S2: IFNARi reduces IFNa-2a-mediated antiviral activity; Figure S3: E2-mediated antiviral effect is ablated by IFNARi; Figure S4: Sex specific gene expression; Table S1: Primer full sequences used for RT-qPCR; Table S2: TaqMan probes used for RT-qPCR.

## Abbreviations

HCV: hepatitis C virus; E2, 17β-oestradiol; ERα/β, oestrogen receptors alpha/beta; GPR30, G protein-coupled receptor 30; IFNARi, IFN alpha-IFNRA-IN-1; IFNα-2a, interferon alpha-2a; MOI, multiplicity of infection; FFU, focus-forming unit; CM, conditioned media; IFNγ, interferon gamma; ERE, oestrogen responsive element; KCs, Kupffer cells; HSCs, hepatic stellate cells; LSECs, liver sinusoidal endothelial cells.
