# Supplementary material for: 17β-Oestradiol Protects from Hepatitis C Virus Infection through Induction of Type I Interferon
Source: Viruses. 2022 Aug 18;14(8):1806. doi: 10.3390/v14081806 (PMC9415988; doi:10.3390/v14081806)
Supplement: Supplementary file 1 [file viruses-14-01806-s001.zip › 20220711 Supp E2.pdf]

Figure S1 - Barbaglia *et al.*

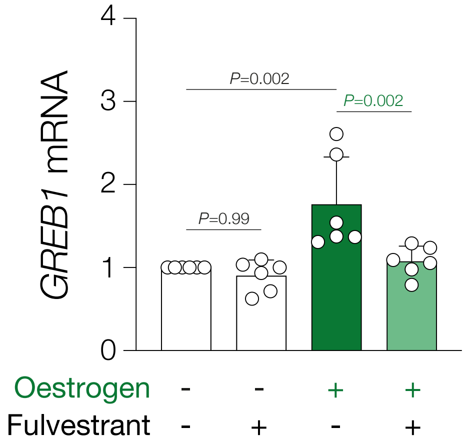

Figure S2 - Barbaglia *et al.*

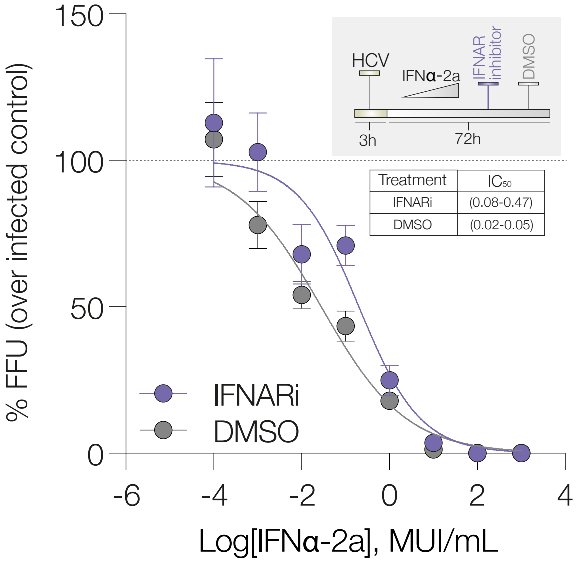

Figure S3 - Barbaglia *et al.*

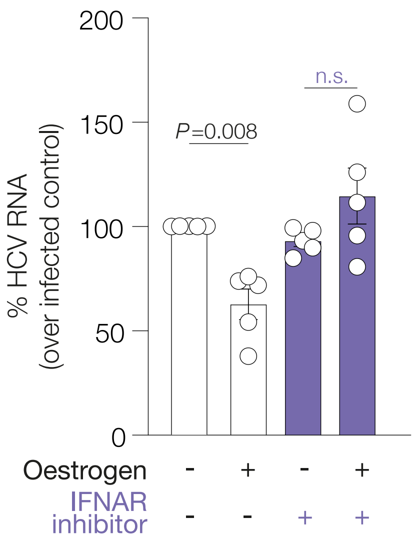

Figure S4 - Barbaglia *et al.*

A

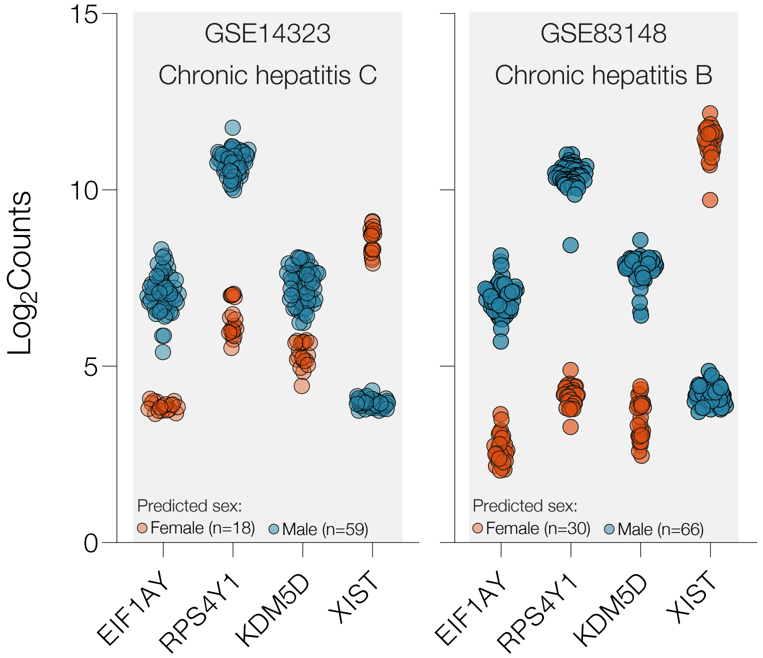

B

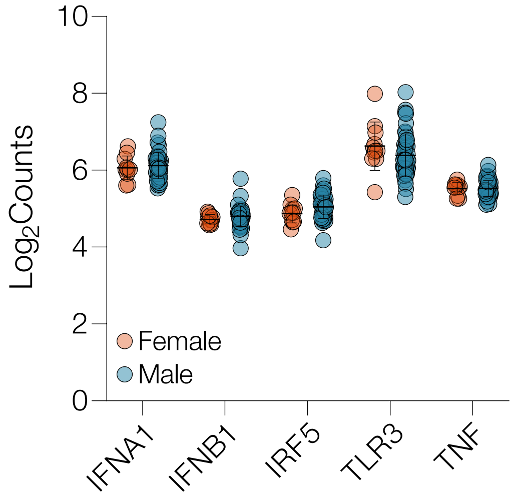

Supplementary Table S2 - Barbaglia *et al.*

Gene	TaqMan Probe	GenBank
EIF2AK2	Hs00169345_m1	AK290655.1
HPRT	Hs02800695_m1	AK313435.1
IFNA1	Hs00256882_s1	AB578886.1
IL6	Hs00985639_m1	A09363.1
ISG15	Hs00192713_m1	BM712238.1
IRF7	Hs00185375_m1	AF076494.1
MX1	Hs00182073_m1	AK096355.1
OAS2	Hs00942643_m1	AK292796.1

Supplementary Table S1 - Barbaglia *et al.*

Gene	Primers
CXCL8	For: 5'-CCAGGAAGAAACCACCGGA -3'<br>Rev: 5'-GAAATCAGGAAGGCTGCCAAG -3'
GREB1	For 5'-GGTCTGCCTTGCATCCTGATCT -3'<br>Rev 5'-TCCTGCTCCAAGGCTGTTCTCA -3'
HPRT	For: 5'-GATTTGGAAAGGGTGTTTAT -3'<br>Rev: 5'-TCCCATCTCCTTCATCACAT -3'
IFIT1	For: 5'-GCCTTGCTGAAGTGTGGAGGAA -3'<br>Rev: 5'-ATCCAGGCGATAGGCAGAGATC -3'
IFNB1	For: 5'-CAGCAATTTTCAGTGTCAGAAGC -3'<br>Rev: 5'-TCATCCTGTCCTTGAGGCAGT -3'
IFNL3	For: 5'-TCGCTTCTGCTGAAGGACTGCA -3'<br>Rev: 5'-CCTCCAGAACCTTCAGCGTCAG -3'
JFH-1	For: 5'-TCCCGGGAGAGCCATAGTG -3'<br>Rev: 5'-TCCAAGAAAGGACCCAGTC -3'
IL1B	For: 5'-ACAGATGAAGTGCTCCTTCCA -3'<br>Rev: 5'-GTCGGAGATTCGTAGCTGGAT -3'
IL18	For: 5'-GACCAAGGAAATCGGCCTCTA -3'<br>Rev: 5'-ACCTCTAGGCTGGCTATCTTTATACATAC -3'
IRF3	For: 5'-ACCAGCCGTGGACCAAGAG -3'<br>Rev: 5'-TACCAAGGCCCTGAGGCAC -3'
IRF5	For: 5'-TATGCCATCCGCCTGTGTCAGT -3'<br>Rev: 5'-GCCCTTTTGGAACAGGATGAGC -3'
TGFB1	For: 5'-CCCAGCATCTGCAAAGCTC -3'<br>Rev: 5'-GTCAATGTACAGCTGCCGCA -3'
TLR3	For: 5'-GCGCTAAAAAGTGAAGAACTGGAT -3'<br>Rev: 5'-GCTGGACATTGTTCAGAAAGAGG -3'
TNF	For: 5'-CCCAGGGACCTCTCTCTAATC -3'<br>Rev: 5'-ATGGGCTACAGGCTTGTCACT -3'
