# Supplementary material for: 17β-Oestradiol Protects from Hepatitis C Virus Infection through Induction of Type I Interferon
Source: Viruses. 2022 Aug 18;14(8):1806. doi: 10.3390/v14081806 (PMC9415988; doi:10.3390/v14081806)
Supplement: Supplementary file 1 [file viruses-14-01806-s001.zip › 20220711 Supplementary Legends.pdf]

Supplementary Figure Legends.

Figure S1. E2 stimulation increases the level of control gene *GREB1*. HuH7 cells were treated for 4h with fulvestrant (second column), 17β-oestradiol (third column) and their combination (fourth column). Cells were then incubated in fresh media and *GREB1* mRNA levels were evaluated at 6h post treatment. Data were expressed as relative mRNA levels normalized to control (first column). Data are reported as mean with SEM of at least 3 independent experiments, with 2 biological replicates. Statistical significance evaluated by Mann–Whitney U test.

Figure S2. IFNARi reduces IFNa-2a-mediated antiviral activity. HuH7 cells were infected for 3h with HCV. Post infection, cells were incubated for 72h with serial dilution of interferon alpha-2a (from 10^3 to 10^{-4} MUI/mL) supplemented with DMSO or IFNARi (500 nM). Antiviral effect of interferon alpha-2a was evaluated by FFU assay. Data were normalized to infected control (dot line) and were presented as mean with SEM. IC50 values were calculated by non-linear least square regression and IC50 significant difference was determined by unpaired t-test.

Figure S3. E2-mediated antiviral effect is ablated by IFNARi. HuH7 cells were treated with 17β-oestradiol or DMSO for 1h, followed by infection in presence of oestrogen. IFNAR inhibitor was added for the whole time of the experiment. HCV RNA quantification was performed at 72h post infection by qPCR. Results were normalized to infected control and represent the mean with SEM of at least 3 independent experiments, with 2 biological replicates. Statistical significance evaluated by Mann–Whitney U test.

Figure S4. Sex specific gene expression. (A) Affymetrix microarray data was interrogated for the expression of previously defined X and Y chromosome genes from chronic hepatitis C (left) and chronic hepatitis B (right) patient cohorts. (B) Expression of the 5 gene signature in male and female HCV infected patients.

Supplementary table S1. Primer full sequences used for RT-qPCR. C-X-C motif chemokine ligand 8 (*CXCL8*), growth regulating oestrogen receptor binding 1 (*GREB1*), hypoxanthine-guanine phosphoribosyltransferase (*HPRT*), interferon-stimulated gene 56 (*IFIT1*), interferon beta 1 (*IFNB1*), interferon Lambda 3 (*IFNL3*), HCV RNA (JFH-1), interleukin -1 and -18 (*IL1B, IL18*), interferon regulatory factor 3 (*IRF3*), interferon regulatory factor 5 (*IRF5*), transforming growth factor beta (*TGFB1*), Tool like receptor 3 (*TLR3*), tumour necrosis factor alpha (*TNF*).

Supplementary table S2. TaqMan probes used for RT-qPCR. Eukaryotic translation initiation factor 2 alpha kinase 2 (*EIF2AK2*), hypoxanthine-guanine phosphoribosyltransferase (*HPRT*), interferon alpha 1 (*IFNA1*), interleukin-6 (*IL6*), interferon-stimulated gene 15 (*ISG15*), interferon regulatory factor 7 (*IRF7*), myxovirus (Influenza) resistance 1 (*MX1*), 2'-5'-oligoadenylate synthetase 2 (*OAS2*).
